# Foreign Summary

**Published:** 1839-06-01

**Authors:** 


### FOREIGN SUMMARY.

*Abernethiana.*—The following amusing anecdotes of Abernethy, the Medico-Chirurgical Review has culled from Mr. Pettigrew's Medical Portrait Gallery.

A man of rank consulted Mr. Abernethy, and was received by him with remarkable rudeness. Upon some severe remark being made, the patient lost his temper and told Mr. A. he would make him *eat his words.* "It will be of no use," said Mr. A. coolly, "for they will be sure to come up again!"

"Pray, Mr. Abernethy, what is a cure for gout?" was the question of an indolent and luxurious citizen. "Live upon sixpence a day and earn it," was the cogent reply.

He is reported to have been consulted by the late Duke of York; and he stood before his royal highness, whistling, with his hands in his breeches-pockets, as usual. The duke, astonished at this conduct, said, "I suppose you know who I am." "Suppose I do," said he, "what of that?" And his advice to his royal highness was given thus: "Cut off the *supplies*, as the Duke of Wellington did in his campaigns, and the enemy will leave the citadel."

A barrister had a small ulcer on the leg which was difficult to heal, and he determined to apply to Mr. Abernethy. Aware of his impatience and eccentricity, he, immediately upon entering his room, began to pull down his stocking. "Halloa! halloa! what the devil are you at?" said the surgeon. "I don't want to see your leg; that will do—put it up, put it up." The patient did so; but justly dissatisfied with the imperfect manner in which his case had been considered, he, instead of the usual fee, placed a shilling only upon the table. "What is this?" said Mr. A. "Oh," replied the barrister, "that will do—put it up, put it up," and coolly walked away.

*Abernethy as a Lecturer.*—The lecture room was the grand theatre upon which Mr. Abernethy displayed; there, indeed, he

"Shone eccentric, like a comet's blaze!"

And there he would indulge his disposition and propensities to an extent which occasioned the pupils frequently to regard it as an exhibition, and called it an "Abernethy at Home." His mode of entering the lecture-room was often irresistibly droll—his hands buried deep in his breeches-pockets, his body bent slouchingly forward, blowing or whistling, his eyes twinkling beneath their arches, and his lower jaw thrown considerably beneath the upper. Then he would cast himself into a chair, swing one of his legs over an arm of it, and commence his lecture in the most *outre* manner. The abruptness, however, never failed to command silence and rivet attention.

"The count was wounded in the arm—the bullet had sunk deep into the flesh—it was, however, extracted—and he is now in a fair way of recovery! That will do very well for a novel, but it wont do for us, gentlemen: for Sir Ralph Abercrombie received a ball in the thick part of his thigh, and it buried itself deep, deep: and it got among important parts, and it couldn't be felt; but the surgeons nothing daunted, groped, and groped, and groped,——and Sir Ralph died."

*Abernethy at the last.*—His eccentricity continued during his existence, and, towards the last, he is reported to have joked upon the œdematous state of his legs, produced by the disturbance of the circulation and his difficulty of breathing. Some one inquired of him how he was? to which he replied, "Why I am better in my legs than ever; you see how much stouter they are!" His hobby retained full possession of him, also, to the end of his life. He attributed his disease to the stomach. He said, "it is all stomach; we use our stomach ill when we are young, and it uses us ill when we are old." But it is not a little singular, that he expressly enjoined that no examination of his body should take place!

---

*Professor Dieffenbach on the Orthopædic Establishments in Paris.*—The great progress which orthopædy has made of late years in France, has attracted the attention of most professional visitors to Paris. Indeed, after lithotrity, the treatment of deformities and contractions has become quite a favourite subject with many of the leading surgeons in that metropolis. Not many years ago, the management of such cases was not deemed worthy of the surgeon's attention; and it was therefore left to corset-makers, and to others equally uninformed, to do the best that they could to rectify, or, at all events, to conceal the deformity. But now there are regular establishments, conducted by most able and intelligent men of education, in the neighbourhood of Paris, to which resort, from all parts of France, an immense number of persons who are afflicted with curvature of the spine, contractions of the limb and such-like maladies. The most celebrated of these are situated at Passy, and belong to M. Guérin, and to M. Bouvier. Both institutions are conducted with admirable skill, and afford to the invalids every advantage which the ingenuity and experience of these gentlemen can afford. The arrangements for gymnastic exercises, for the use of extending machinery and bandages, for the improvement of the general health of the patients, and, at the same time, for the recreation and instruction of the mind, are most complete in every particular.

In cases of curved spine, the extension is effected by means of a peculiarly constructed bed to which the patient is secured, and the length of which may then be increased or diminished as the surgeon may require. The one used and recommended by M. Guérin consists of four different segments or smaller beds, which can be separated from each other, or screwed together at pleasure. By moving one or more of the pieces to either side, after the patient has been secured by the bandages and straps, a certain lateral direction may be given to the extending force, so as to draw the spine either to the right or to the left hand, according to the inclination of the curvature. The peculiarity of M. Guérin's extending bed is its being composed of several distinct pieces. The entire establishment of this gentleman, at Passy, deserves the highest praise. The great Monthyon prize was, some years ago, awarded him by the Royal Academy at Paris, for the various improvements which he had introduced into the scientific practice of Orthopædy. His museum contains numerous specimens of comparative as well as human anatomy, and also a most instructive collection of Paris-plaster casts of the various forms of distortion and irregularity of the spine and joints, at various periods of their treatment.

The employment of gymnastic exercises holds a prominent place in the orthopædic practice of

M. Guerin. In the great hall of his establishment are to be seen all sorts of machines and apparatus for the use of the inmates. Swimming, and other exercises in which the body lies as in swimming, are particularly recommended, both by M. Guerin and M. Bouvier. Another exercise, which is very generally practised by young invalids, is going on tall crutches. "I was," says Professor Dieffenbach, "quite astonished, upon entering the gardens, to see a number of French girls moving about with surprising agility upon tall crutches, like so many kangaroos with their short arms and long legs." The staves of the crutches are very tall, so that when the invalids stop, their feet do not touch the ground, but are suspended, *schwebend in der luft*, half an ell above it, and the body is supported by the arms.

Professor Dieffenbach seems to approve of this mode of exercise, and recommends it to his countrymen for imitation. He mentions with praise the collection of casts and other illustrative preparations in the museum of M. Bouvier, and alludes in particular to one cast, to which M. Bouvier directed his attention, as being quite "une moule historique." Its history is rather curious, and may be instructive as well as interesting to the reader.

An orthopædist of some eminence in Paris, announced, some time ago, to the Institute of France, that he had discovered a method of removing curvatures of the spine in a very short period of time; and, with the view of convincing the members of the truth of his claim, he brought before their notice a young woman in whom there was a very marked irregularity of the spine, which he undertook to rectify in the course of a few weeks. A commission was appointed to investigate the subject. Paris-plaster casts were taken of the patient's back, and every other means to arrive at the truth were adopted.

Within a very short time, the girl was again presented before the Institute, and now not the slightest trace of deformity was visible. The commissioners were quite satisfied of the identity of the individual, and could not help expressing their astonishment at the extraordinary rapidity of the cure; it seemed to them quite miraculous. M. Guerin, however, suspected that there must be some deception, and that the case was, in all probability, one of simulation.

He soon convinced the commissioners of the truth of his suspicions, by proving to them, that although the line of the spinal column seemed to be quite as much deformed in the simulated as in the genuine case of disease, the muscles of the back were at the same time dislocated in the latter, but not in the former instance. By attending, therefore, to the condition of the spinal muscles in a suspected case, we may generally detect when a fraud is attempted to be played upon our judgment.

The zeal with which M. Guerin exposed the impudence of the above trick, so irritated the impostor, that he brought an action at law against his adversary; and, although all right and equity were on the side of M. Guerin, he was amerced in a heavy penalty for the injury he had done the plaintiff's character.

Professor Dieffenbach pays a high compliment of admiration to M. Bouvier, for the pleasure and instruction he had received from visiting his orthopædic establishment; and he alludes, with great satisfaction, to his having met there some of the ablest professional men in Paris, such as the two Larreys, father and son, Marjolin, Le Roy, and many others. The second prize of 2,000 francs has been awarded, by the Institute, to M. Bouvier, for the various improvements which he has introduced.

For some time past, he has had the charge of all cases of deformity in the Hôtel Dieu, and, also, in the Hôpital des Enfans Malades; and at these two great institutions the medical students have an opportunity of acquiring a scientific knowledge of orthopædy.

Besides the establishments noticed above, there has been for some time a sort of ambulatory infirmary for the treatment of deformities in Paris; this is under the management of MM. Bouvier and Duval. At a stated hour each day a great number of cases, among children chiefly, come for advice, bandages, and so forth—all which are given gratuitously. M. Duval attends to the cases of club-foot, and M. Bouvier to those of spinal deformity. In lateral curvatures of the back, among the poor, and those who cannot be treated at home with the extending bed, M. Bouvier employs the girdle or cincture invented by M. Hossard, (ceinture à levier ou inclinatoire,) by means of which the depressed shoulder is elevated, and the spine is inclined somewhat over to the side opposite that of the curvature.

Many of the cases of club-foot are treated by the section of the tendo-Achillis, as recommended by M. Stromeyer.—*Medico-Chirurg. Rev. for April, from Zeitschrift furdie Gesam. Med.*

---

*On Idiopathic Irritable Bladder.* By F. Hale Thomson, Esq., Assist. Surgeon to the Westminster Hospital.—Mr. Thomson delivered, this day, the first of a series of clinical lectures. As an exordium, he made several observations on the importance of clinical instruction and note-taking. General lectures on the different branches of the healing art afforded but an outline; but demonstrations of actual disease filled up the details of the picture, and gave the student a practical acquaintance with diseases. Note-taking, he said, was of more importance than was generally imagined; it not only contributed to make the knowledge of the student more precise and circumstantial (and no knowledge was useful which was not exact,) but furthermore, it tended to form in the mind habits of accurate thinking, which were not only useful in the acquisition of science, but also in the ordinary business of life. The subject chosen by Mr. Thomson for his first lecture was, "idiopathic irritable bladder," a malady, unfortunately, not of uncommon occurrence. The bladder, he said, was an organ possessing very extensive sympathy with the states and conditions of the other viscera. An irritable state of the brain affected, speedily, the functions of the bladder; and even certain mental affections, as was evident in the case of fear or anxiety, where not only an increased secretion from the kidney occurred, but the bladder, though not distended, became impatient of its contents. But injuries, and maladies affecting the head, were not the only causes which disturbed the functions of this important organ. Mr. Thomson had known instances in which compound dislocation of the ankle had produced irritable bladder.

It was obvious that the functions of the bladder must be unfavourably influenced by the presence of organic disease, in its own texture, or in contiguous and continuous structures; for example, by enlargement of the prostate gland; by disorganised kidney; by the accretion of calculi; by tumour, inflammation, or ulceration of adjacent organs. The diagnosis of these structural diseases was not involved in any great difficulty; the most obscure, perhaps, was Bright's disease of the kidney; and this could be satisfactorily ascertained by the use of a few simple reagents; for example, merely elevating the temperature of the urine of a patient affected with this malady to 150° Faht. would coagulate the albumen, which is characteristic of it, and make evident numerous particles of *cruorin* diffused through the serum.

The disease, however, to which Mr. Thomson wished particularly to draw the attention of the students was simply a functional derangement of the organ, in which there existed no alteration of structure either in itself, or in any other of the pelvic or urinary apparatusses. This disease had been well illustrated in the case of Robert Stodart, a man about forty years old, and who had been seen by all the gentlemen present. This patient was by trade a tailor, but had been at sea for several years. When admitted into the hospital, on the 12th Feb., 1839, he was exceedingly emaciated; he had incontinence of urine, attended with great pain on voiding it, and for some time afterwards. He had been suffering more or less from this inconvenience for the last two years; but for nine months previous to his admission he had been totally unable to retain his water either night or day. He acknowledged to have led a very debauched and irregular course of life, and to having freely indulged in inebriety. Until within the last two years, however, his health was not apparently much impaired. Two years ago he was, according to his own statement, suddenly seized with rigors and pain in the back; he applied to a neighbouring dispensary; he was bled from the arm and cupped on the loins, and in a few weeks he convalesced. At this period he observed, for the first time, that his urine passed in a forked stream. About July or August last, he found that his urine flowed in unusually small quantities, and that micturition was attended with considerable pain. These symptoms gradually increased in intensity, till he was reduced to the most distressing state from the constant involuntary dribbling of the contents of the bladder. He had had two or three severe attacks of gonorrhœa, and had for some time found the sitting posture uneasy to him. On the day after his admission, (13th Feb. ult.,) Mr. Thomson prescribed for him the late Mr. Lynn's favourite aperient draught, viz., half an ounce of castor-oil, mixed with the same quantity of tincture of senna. A silver catheter was introduced into the bladder, but not without some obstruction at the prostatic part of the urethra. A large quantity of dark-brown, and highly ammonical urine, was drawn off, and a considerable quantity of mucus followed. Upon testing the urine, it was found to be alkaline; the bladder was injected with warm water, and the following medicine exhibited:—

*Phosphoric acid*, ʒij.;
*Decoction of pareira brava*, f℥viij. An ounce twice a day. Lynn's draught every morning, and ten grains of Dover's powder every evening. He was placed on a fish diet.

Feb. 15. He had passed a good night, had perspired profusely, and had felt no pain since the bladder was injected with warm water. The urine had passed three times in a natural manner.

16. The bowels being confined, he was purged with chloride of mercury and extract of colocynth and a senna draught.

17. The man was much improved. The urine drawn off exhibited no alkaline properties.

18. The bowels became constipated again, and a good deal of irritative fever was excited, in consequence of which the catheter was not introduced this day. He was purged with jalap and senna, and a febrifuge mixture was resorted to, consisting of acetate of ammonia, antimonial wine, and camphor julep.

19. Having much pain in the loins, he was ordered a strengthening plaster.

20. The medicine had induced nausea and vomiting, but the irritation of the bladder was diminished. An effervescent mixture ordered.

21. The urine being tested was found to be acid, and doses of carbonate soda were consequently given with aperient medicine.

25. To this day the condition of the patient was improved; but he now experienced a relapse into irritative fever, for which the same remedies were prescribed as in his former attack.

26. The fever had subsided; the urine had improved in quality, and was retained for longer periods.

27. Much better. From this period up to the 7th March he steadily improved, when he was allowed mutton chops for his dinner.

On the 10th March, No. 11 catheter was passed into the bladder, and the urine extracted was perfectly healthy. He had, on the 20th March, lost all pain in the loins; he passed a full stream of urine without pain, and his general health was perfectly re-established.

The species of irritable bladder from which Stodart had suffered, Mr. Thomson said, was not unknown to authors: Sir Everard Home, Sir Benjamin Brodie, and Mr. Abernethy, were the best authorities, and all of them had experienced difficulty in defining the etiology of the disease. There existed, therefore, some ambiguity in the acceptation of the term "irritable bladder." It was to be distinguished carefully from the symptomatic irritation arising from organic disease of the prostate, urethra, or kidney, as well as from acute inflammation of the bladder, where the treatment would, of course, be materially different. The disease exemplified in the case of Stodart, might depend upon various causes; it might depend upon a peculiar quality of the urine; it might depend upon a peculiar state of the nerves of the organ in which the sensibility was augmented, and the motor power enfeebled. Mr. Abernethy used to call this state of bladder "an undefinable state of the nervous functions." This was simply an acknowledgment of ignorance upon the subject, and was, perhaps, more useful than the promulgation of an ingenious but delusive theory.

Sir Benjamin Brodie stated that this affection was common in men of about fifty years of age, and was accompanied, generally, by an obstinate headach, which was occasionally mistaken for the primary disease, and mischief was consequently done through injudicious treatment. It would be necessary, when patients of that description complained of headach, to ascertain the condition of the urinary organs. Men in good circumstances at this time of life, having lost their zest for more active pleasures, often addicted themselves to the seductions of the table, and indulged their appetites to an inordinate extent. The powers of digestion were consequently injured, and the bladder was the first organ to sympathise with the general depravity of the health.

The first task of the surgeon was to diagnosticate between this disorder when arising primarily from the state of the bladder, and when arising from the presence of concretions in the kidney, or in the viscus itself: and from affections of the organs most intimately connected with the bladder. Acute inflammation of that viscus might be known by the severity of the symptoms: calculous concretions in the kidney, by the wasting and enduring hectic, and a careful application of the numerous diagnostic means devised within the last dozen years, could not fail to lead the judicious surgeon to a just appreciation of all the morbid changes affecting these parts.

The treatment of irritable bladder will be modified by the varying circumstances of each case. In general the diet must be plain and nutritive, but not stimulant. The patient should be placed often in the horizontal posture, but he should also have regular, though gentle, exercise. The emptying and periodical washing out of the affected viscus were of primary importance. The alimentary canal should, of course, be regularly cleaned out. When the urine was alkaline, no medicine was so effectual as the phosphoric acid, given in doses cf ten drops, three or four times daily. This might be combined advantageously with the decoction of the pareira brava, which he (Mr. Thomson) had found a most excellent tonic. After taking this combination for two or three days, the patient generally found the quality of his urine greatly improved, and the muscular power, both of detrusor and sphincter, augmented. He had always found the phosphoric acid much superior to the mineral acids. When the urine was acid, the carbonate of soda, in scruple doses, was an admirable remedy, and, according to his experience, very superior to potass, although that alkali was recommended by the high authority of Dr. Prout. As auxiliary means in the treatment of irritable bladder, Mr. Thomson spoke very highly of copaiba, uva ursi, and colchicum. These were, also, very efficient alleviants in the symptomatic affections of that organ. Copaiba, especially in enlarged prostate, often afforded signal relief, by increasing the mucous secretions of the urethra. He could not concur in the praises which Sir Benjamin Brodie had bestowed on turpentine. In some constitutions that medicine aggravated all the affections of the urinary apparatus, and he (Mr. T.) had known instances where this medicine, when used for other maladies, had actually brought on the disease for which it was recommended as a remedy. In symptomatic affections of the bladder, all the symptoms of idiopathic irritable bladder might exist, and, therefore, in these cases, the application of the various remedies he had suggested would be highly proper, though they might not be effectual in curing the patient. Phosphoric acid would be always proper, when it was necessary to correct the alkalinity of the urine, and carbonate of soda, where acidity was the mischief to be removed; and in all chronic cases, whatever added to the vigour of the general health must be conducive to good.

The occurrence of irritative fever was a circumstance often embarrassing to the surgeon; it arose apparently from catheterism, and it became necessary to desist from drawing off the urine of the patient. When the symptomatic irritative fever was removed, by the means already designated, the catheterism might be resumed. The case of Stodart afforded an excellent example of what might be effected by very simple means, when carefully and judiciously applied; nothing could afford a greater contrast than the actual state of the patient, compared with his state on admission, on the 12th of last February. His general health was then at the lowest ebb, and every night his bed-clothes were saturated with urine; but now the tonicity of the bladder was restored with the general tone of the system.

Mr. Thomson now exhibited an injecting syringe and several catheters constructed by Weiss. For the principle of these improved instruments we were indebted, he said, to the inventors of *lithotripsy*. Surgeons should be particularly careful to have the syringe made so as to suit the size of their own hands: the length of the piston should be adapted to the degree of expansion which the surgeon could effect between his fingers and his thumb; so that, having two fingers fixed in rings on each side of the piston, and his thumb in a ring on the top of the piston, he should be able so to expand his hand as to draw the piston completely up, by means of one hand only.—*Lancet.*

*Parisian Medical Sketches—Sœurs de la Charité.*—What can I say good enough of you, ye good sisters of charity! Rather than write common place encomiums on virtue like yours, I would be silent altogether; yet how can I be silent who have been so often moved by your steady enthusiasm, and by the almost daily exhibition of some act of virtue, of which your whole lives are but the history! Ye unpaid nurses of sorrow and of sickness, whose only supports and comforters ye are—who, in the midst of "all the sad variety of woe," when all medication fails, and life is fast ebbing away, still abide by the dying, and, when all is over, can go from the dead man's couch undismayed, to smile with a renewed interest on him who is recovering—ye, who practise unheard of self-denial—who never answer the coarse coarsely, or offend the peevish! I have seen ye for years, in many a trying strait, the peace-maker between the refractory patient and the not always reasonable doctor. Come into the ward St. Laureant with me. If you ask why this ward particularly, I will tell you. It is the most revolting ward at St. Louis, and St. Louis is the least enticing of hospitals. Internal disease may, to the patient, perhaps, be even more awful in results, or fears of results, but to the mere spectator, disease can never manifest itself more shockingly than when its ravages are on the surface of the body. No, you will not enter that low-roofed, spattered, and bemired apartment, of which the very look from the door-way terrifies. Why should you breathe that rank, close air, heavy with the smell of sores, of salves, and of sulphur? Why should you penetrate those foul recesses, and pass from bed to bed amidst yon crowd of half-naked ruffians, steeped in abominations of all sorts—youths who speak but to imprecate, and *blasés* old villains, offensive to every sense? To witness, but for once, such a mixture, of fierceness and of filth, of misery united to mischief, you will not, even for curiosity's sake, pass under those low arches; and you are right: mere curiosity must turn away her head, sick, and unable to proceed. Yet, in the midst of all this, which you see from the door that I have opened, behold, and with reverence, one of those modest "sœurs," in her clean white and black head-gear, ministering with her soft, womanly hands to that unsightly crew, or quietly knitting or sewing—for she never permits herself to be unemployed. "Aussi, ce n'est pas par intérêt que nous le faisons," said she to me the other day, smiling in sweet complacency at my unreserved communication of my feelings; which words, I remember, were scarcely out of her mouth, when a refractory young *galeux*, who had been repeatedly admonished, that, if he did not conduct himself better, he would be sent away from the hospital, was summoned to appear before the physician; having listened with impatience to whose reprimand, the monster replied, with great vehemence, and with knit, vindictive brow, "C'est la haine qui fait que la sœur s'est plain de moi!" The sister said nothing, but bent her eyes on the ground; for ourselves we could, even at the chance of catching his disorder, have felled the young recreant with a blow; but the physician's words settled, in a most eloquent manner, this vindictive attack. "Silence, sirrah! and for shame." "Ici on ne reconnait que bienveillance et misericorde!"—out with his name—"rayez son nom!—exeat." Dr. Cazenove was that physician.

Nor are the amiable sœurs' duties confined to such as, it is to be hoped, generally appreciate their inestimable support! What, though they have never been mothers—go to the Hôpital Jesus, filled with sick babies and scrofulous childhood, and say there if it be possible for even maternal στοργη to be more adequately represented. The sick child is a heavy burthen to the fondest parents. We love to play with our brats, which requires them to be well; and more than playthings, till there be some exchange of mind—some companionship—they cannot be. Instinct attaches, and pride is gratified where grace or comeliness qualifies its object: but the sœur looks throughout upon these poor children in a widely different light. With her these hapless little creatures are sacred deposits, of whose souls and bodies alike she considers herself to have become the temporary guardian; and whether it be to flourish on earth, or to be forthwith transplanted into heaven, her tender culture of the young plant is still the same. Any morning between nine and ten, if you ask for the "salle des teigneux," you will find, as I have done, a semicircle of little urchins from seven years up to fourteen, all in their white night-caps, and all but one, who is the biggest, sitting under her presidency to pick lint: he is standing, to read aloud for the benefit of the younger community, while the kind "sister of charity" sits by correcting his blunders; and so intent are the little folk at once on their rag picking and the story, that you may generally approach quite close to them before you are perceived: then, perhaps, an immediate stir takes place; the reader puts his book down disconcertedly, and his kind prompter rises to meet you. Do not now be afraid of putting her out, or scrupulous not to ask her too many questions. The only compliment you can pay a sœur is to take an interest in what most interests her—doing good. If, in showing you her young charges, she does not use periphrasis—if she speak the naked truth sometimes too plainly for your habits, remember the sœur is no fine lady; hers is only the refinement of mind, which religion can alone confer; she knows nothing of conventional phrases. If she conduct you to a poor little thing, frightfully emaciated, whose tense abdomen is blown out like a balloon, and whose attenuated members look rather like a roll of Herculanean papyrus than human skin; and if she quietly state, in his hearing, that he has been given up, and will not live many days, be not shocked without necessity, for the little unconscious creature has none of your fears of dying; he hears the announcement without anxiety or even interest, and asks "sa bonne sœur," with a ghastly effort at a smile, to give him a little drink!—But I am becoming garrulous, and had nearly forgotten, in the society of the good sœurs, that I had a reader, perhaps a fastidious one.

*Doctors.*—If it be true that troopers are extraordinary liars, M. —— would deserve to be their captain. When he chances to be in a good lying humour, it is extremely difficult to keep pace with him. His romances are as long as Scudery's, and much more diverting. I never knew a man so at home in the absurd or the impossible as M. ——. I have seen him at his hospital at 9 A. M., and he has already seen a hundred patients, and been three times round Paris.

Some men are great talkers upon one favourite subject,—he is equally voluble upon all. I have heard him, in the brief space of ten minutes, touch upon literature and lepra, and descant on politics and prurigo. He is addicted—

> "To raising questions dark and nice,
> To solve 'em after in a trice;
> As if Philosophy had catch'd
> The itch, on purpose to be scratch'd."

"Now, gentlemen," he would say, when the visit was over, and he was in the act of untying his apron strings, "now what would you do in such a case as the following?"—and then he would give one in which a conjuror's, not a doctor's, services seemed wanted; such a case, however, had only just happened to him, and was already doing well. The patient was generally an Englishman, "grand bel homme (fort distingué du reste,") whose name he was not permitted to mention. Of what he told us while he was washing his hands, and we were looking for our hats, the following is a *souvenir*:—"Ecoutez!"—we turned our heads; "I am going to put a case to you—or, stay, I may as well tell you the case, as it occurred to me some weeks ago. An Englishman had an angry discussion with his wife; he determined, therefore, to commit suicide, and divided his throat from ear to ear with a razor. I was called in. I never saw any thing so frightful in my life: blood was pouring from an immense gaping wound all round the neck; besides which he had lost a *mer de sang* before I saw him! Having satisfied myself as to the cause of this rash act, I requested my patient (as I saw no time could be lost) to let me stay the bleeding. 'Sir,' replied the man, (whose throat was cut from ear to ear,) 'I am weary of life—(le *tædium vitæ* des Anglais) let me die.' But having lectured him upon the impropriety of his conduct, and having represented to him, 'sa position, ses devoirs,' &c., he at length consented to my saving him; adding, however, that the attempt was quite futile—that I might spare myself the useless trouble—that he had cut his throat too deep, and that nothing could now succeed." The philosophy of Seneca in the bath was a joke to the *sang froid* of our distinguished countryman. "What did you do, sir?" "Il n'y avait rien de plus simple. I contented myself, having drawn down his head, with keeping the divided surfaces together with a few simple stitches; requesting him to repose, and not, upon any account, to turn his head. He complied with my request, and, at the end of five days, I had the pleasure of meeting him on foot, on the Boulevard Italien." "The treatment was very simple," said the youthful clerk. "Yes, when you know it," said the other: "but, gentlemen, allow me to put to you a less serious case, and see how you could extricate yourselves under the following circumstances:—Suppose yourselves, any of you, summoned to a great lady, living in a great house at the fashionable quarter of Paris. She is extremely sensitive and impressionable; she has snapt a needle in her finger, which lies at some depth from the surface, between it and the nail. I know what you will say—'I would get a pair of English tweezers,' *bien fines*, 'and try to draw it out; or, if too low, I would make a little incision, and then use the tweezers.' Ah, no! at least, that is not what I did. Guess again. Come, I'll tell you what you would have done on reflection, and it is just what I did: I ordered a basin of warm water, and made the lady soak her hand in it till the nail began to soften; then I took a very fine penknife, and paring away layer after layer of the horn, it became at last as thin as silver paper. Then I made an easy aperture through its side, right over the needle, which I now drew out without the least difficulty, and without giving the lady any pain, which was what I particularly desired to avoid. Tenez, voilà encore un cas lequel s'est presenté depuis peu." This was the case of a fish-hook, which had penetrated deeply into the hand;—but I did not stay to hear whether it was of English or Irish manufacture, of the Limerick or Kirby bend; nor whether nor how it was extracted.—*London Med. Gaz.*

---

A new weekly medical Journal has been started in Dublin, "the Dublin Medical Press." The demand for the *weeklies* increases.

---

*Pathology of Porrigo.*—The observations of Bassi and Audouin on the nature of muscardine—a disease to which silkworms are subject—had proved that it was owing to the growth of minute fungi on the animal. Professor Schoenlein, of Zurich, has been led to examine, under the microscope, some cutaneous eruptions. On the first examination of a pustule of porrigo lupinosa, he satisfied himself of the vegetable and fungous nature of the pustule. Professor Schoenlein is busily employed in prosecuting this subject, and means soon to publish the results of his investigations.—*Müllers Archiv., from Lond. Med. Gaz.*

